# Furostanol Saponins and Ecdysteroids from Plants of the Genus *Helleborus* as Phagostimulants and Predator Deterrents for Larvae of Two *Monophadnus* Sawfly Species

**DOI:** 10.3390/plants13162230

**Published:** 2024-08-12

**Authors:** Jose M. Prieto, Alison M. Barker, Urs Schaffner, Joëlle Quetin-Leclercq, Alessandra Braca, Jean-Luc Boevé

**Affiliations:** 1 Department of Pharmacy, University of Pisa, 56126 Pisa, Italy; alessandra.braca@unipi.it; 2 CABI Switzerland, 2800 Delémont, Switzerland; alisonmbarker@aol.com (A.M.B.); u.schaffner@cabi.org (U.S.); 3 Pharmacognosy Research Group, Louvain Drug Research Institute, UCLouvain, 1200 Woluwé-Saint-Lambert, Belgium; joelle.leclercq@uclouvain.be; 4 DO Taxonomy and Phylogeny, Royal Belgian Institute of Natural Sciences, 1000 Brussels, Belgium; jboeve@naturalsciences.be

**Keywords:** Phymatocerini, Ranunculaceae, chemical ecology, phagostimulation, sequestration

## Abstract

Sawfly species of the genus *Monophadnus* are specialised on Ranunculaceae plants from which the larvae can sequester furostanol saponins into the haemolymph, mainly (25*R*)-26-[(α-L-rhamnopyranosyl)oxy]-22α-methoxyfurost-5-en-3β-yl-*O*-β-D-glucopyranosyl-(1→3)-*O*-[6-acetyl-β-D-glucopyranosyl-(1→3)]-*O*-β-D-glucopyranoside (compound **1**). In this work, TLC, GC-MS, and HPLC-DAD-ESI/MS analyses together with feeding, repeated simulated attacks, and ant deterrence bioassays were conducted to extend the chemoecological knowledge about two sawfly species specialised on *H. foetidus* L. (*Monophadnus* species A) and *H. viridis* L. (*Monophadnus* species B). Larvae of *Monophadnus* species B were mostly feeding on the squares treated with the *n*-butanol fraction from *H. foetidus*, compound **1** being its primary non-nutritional stimulant. In contrast, all *H. viridis* fractions stimulated feeding, with *n*-hexane marginally more active. β-sitosterol within *n*-hexane was determined as the nutritional stimulant. Quantitative analyses demonstrated that leaves of *H. viridis* but not *H. foetidus* contain the ecdysteroids 20-hydroxyecdysone and polypodine B. Moreover, the haemolymph of *Monophadnus* species B larvae reared on *H. viridis* contained the glycosides of polypodine B and 20-hydroxyecdysone at a concentration of 2.5 to 6.8 µmol/g fresh weight of haemolymph. This concentration is several thousand times higher than the concentration range of the aglycones in their host plant (3.63 × 10^−4^ to 2.23 × 10^−4^ µmol total ecdysteroids/g fresh weight of leaves), suggesting bioaccumulation. The larvae of both species fed on *H. foetidus* do not show any traces of ecdysteroids in their haemolymph, indicating a facultative role of these compounds in their defence as well as their inability to endogenously synthesise these compounds. The haemolymph containing ecdysteroids was a significant feeding deterrent against *Myrmica rubra* L. ant workers (one of their natural predators) at 0.8 mg/mL. The larvae kept effective deterrent levels of glycosylated ecdysteroids (≅175 mM) between simulated attacks on days 1 and 2, but the levels clearly decreased on day 3 (≅75 mM). Most larvae (89%) survived a first attack but only 23% a consecutive second one. As a conclusion, we report for the first time that two *Monophadnus* species feeding on *H. viridis* sequester phytoecdysteroids into the larval haemolymph in the form of glycosides. In addition, compound **1** possesses defensive and phagostimulant activities, and we present evidence for a combined effect of furostanol saponins and ecdysteroids as repellents against ants.

## 1. Introduction

Plants face a constant threat of being eaten, driving the evolution of diverse defence strategies. One such strategy is “becoming poisonous”, i.e., the biosynthesis of secondary metabolites toxic to herbivores [1]. There is indirect evidence that poisonous plants communicate their toxicity to animals, largely achieving successful co-existence, as long as herbivores can choose to eat other non-toxic plants [2].

Driven by the selective pressures of competition and predation, numerous herbivorous insects have undergone dietary niche differentiation by specialising on toxic plants. This shift offers a dual benefit: Firstly, it reduces competition from non-specialised herbivores. Secondly, the toxicity of the host plant provides a form of associational defence against both dedicated predators and larger herbivores that might inadvertently consume the insects alongside their fodder [3], although some insect species are themselves toxic to cattle [4]. Furthermore, some insects also reuse harmful plant compounds to their own benefit in what is known as “sequestration”, the selective uptake and storage of plant allelochemicals (toxins) for defence [5]. However, detoxification or excretion are also possible strategies [6]. Notably, herbivores and non-herbivores can synthesise de novo defence chemicals which are similar to those produced by plants (“convergent evolution”) [7].

**Figure 1:**
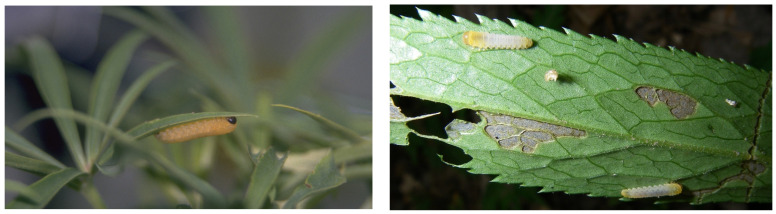
Larvae of *Monophadnus* species A (**left**, picture by Alison M. Barker) and *Monophadnus* species B (**right**, picture by Jean-Luc Boevé) attached to the underside of a leaf of *Helleborus foetidus* L. and *Helleborus viridis* L., respectively.

**Figure 2:**
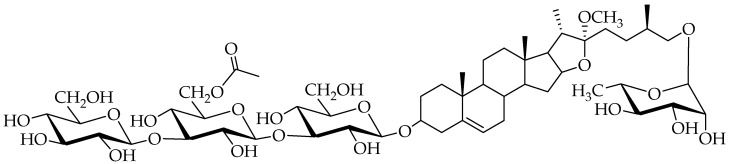
Steroidal furostanol saponin (25*R*)-26-[(α-L-rhamnopyranosyl)oxy]-22α-methoxyfurost-5-en-3β-yl-*O*-β-D-glucopyranosyl-(1→3)-*O*-[6-acetyl-β-D-glucopyranosyl-(1→3)]-*O*-β-D-glucopyranoside (compound **1**) found in *H. viridis*, *H. foetidus*, and the haemolymph of the two *Monophadnus* spp. that feed on them.

Sawfly larvae of the tribe Phymatocerini (Hymenoptera: Tenthredinidae: Blennocampinae) are generally specialised on toxic plants of the orders Liliales and Ranunculales [8]. Several plant species within these orders account for a significant number of poisonings in cattle [2]. Previously, we described how phymatocerine *Monophadnus* larvae (Figure 1) sequester a steroidal furostanol saponin (compound **1**; Figure 2) present in Ranunculaceae leaves into their haemolymph, the blood-like fluid of invertebrates [9], and how the haemolymph is exuded by local disruption of the integument and release of haemolymph droplets upon a predator’s bite [10,11,12]. This defence strategy has been called “easy bleeding”.

**Figure 3:**
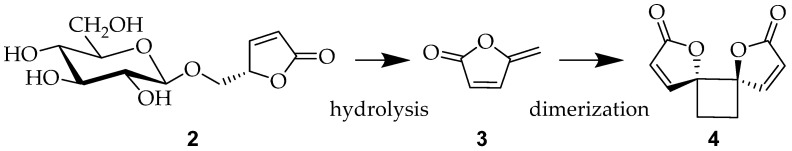
Ranunculin (compound **2**) and its derivatives protoanemonin (compound **3**) and anemonin (compound **4**) are characteristic g-lactones found in *R*-type Ranunculaceae.

**Figure 4:**
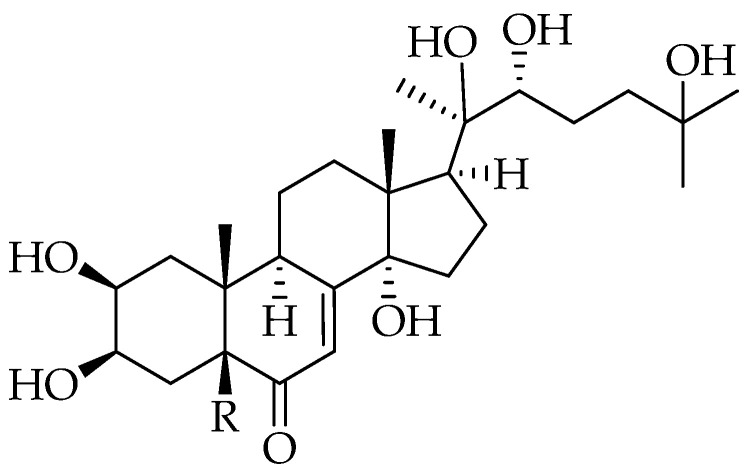
The ecdysteroids 20-hydroxyecdysone (R = H) (compound **5**) and polypodine B (R = OH) (compound **6**) are naturally occurring in *Helleborus viridis* but not in *Helleborus foetidus*.

Compound **1** is a feeding deterrent against ants (one of their natural predators) at the concentration found in the larval haemolymph of two *Monophadnus* species (at least 1.2 μmol/g FW) [9]. Although this compound seems to play a major role in the chemical defence of *Monophadnus* larvae, other plant secondary metabolites found in the *R*-type Ranunculaceae such as γ-lactones derivatives [13] (Figure 3) and ecdysteroids [14] (Figure 4) may also be involved in the chemoecological relationship between *Monophadnus* larvae and their host plants, mainly of the genera *Helleborus*, *Ranunculus, Clematis*, and *Pulsatilla*.

Insects that acquire defensive chemicals (allelochemicals) from their host plants might benefit evolutionarily from recognising these plants, at least partially, by their chemical cues [15]. This idea is supported by research showing that the steroid alkaloid which is sequestered by the larvae of *Rhadinoceraea nodicornis* (Konow, 1886) also acts as a phagostimulant for the larvae [16].

Therefore, we here aim to investigate whether sawfly species of the genus *Monophadnus* specialised on plants of the genus *Helleborus* sequester additional plant secondary metabolites besides compound **1** into their larval haemolymph. We further want to explore whether these sequestered metabolites act as feeding stimulants (phagostimulants) for the larvae and as deterrents against predators. The results of this study will serve to better understand the chemoecological relationship of *Monophadnus* larvae with their host plants (*Helleborus* spp.) and possibly extend the discussion to other sawfly species with similar defensive “easy-bleeding” behaviour.

## 2. Results

### 2.1. Comparative Chemical Analyses of Host-Plant and Larval Haemolymph Extracts

The haemolymph of the different larval samples revealed considerable variation in the types of metabolites present, as shown in Table 1. All samples contained saponins; in addition, *Monophadnus* species A had sterols whilst *Monophadnus* species B had ecdysteroids in their haemolymph. The types of metabolites detected in the haemolymph of the two *Monophadnus* species were also found in their respective host plants suggesting potential bioaccumulation processes.

### 2.2. Composition of Essential Oil from Helleborus spp. Leaves

Overall, the essential oils of *H. viridis* and *H. foetidus* consisted of a high proportion of unidentifiable sesquiterpenoids and diterpenoids (Table 2). The profile of volatiles in the leaves of *H. foetidus* L. was more complex than the one in *H. viridis*, where only a few of them were identified.

We observed in both samples the presence of a peak with a 96 *m*/*z* base peak at 4.2–4.3 min which was assigned to protoanemonin (compound **3**, C_5_H_4_O_2_; 96.08 Da) in accordance with the literature data [17]. The identity of this peak was confirmed by injection of the synthetic standard (Section 4.2). This compound was found in traces in other *Helleborus* [17,18].

*Helleborus viridis* and *H. foetidus* also shared the presence of benzeneacetaldehyde and (*E*)-2-Hexenal. Hexenals and hexenols are released as a result of lipoxygenase activity when leaves are damaged mechanically (i.e., cut prior to hydrodistillation) [17]. The result is an enrichment of the essential oil in (*Z*)-3-hexenol and (*E*)-2-hexenal, popularly known as “leaf aldehydes”. They are known to have important chemoecological roles in insect–plant and insect–insect communication [19,20].

### 2.3. Phagostimulant Activity of Plant Extracts, Fractions, and Isolates in Monophadnus spp. Larvae

**Figure 5:**
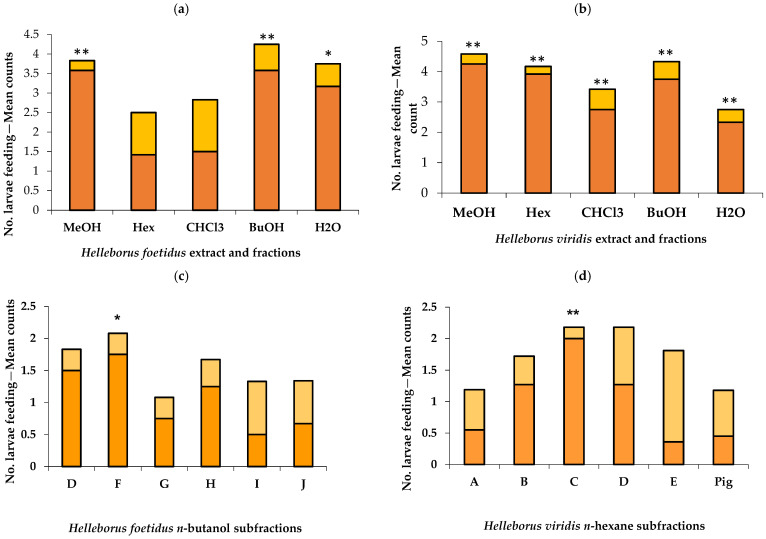
Feeding bioassays of *Monophadnus* species B on paper squares treated with the initial crude methanol extract and its increasing polarity solvent fractions (**a**,**b**) and selected subfractions of the active *n*-butanol fraction of *Helleborus foetidus* (**c**) and the *n*-hexane fraction of *Helleborus viridis* (**d**) leaves. Orange and yellow colours indicate larvae feeding on fraction-treated and solvent control squares, respectively, at 6 h. (Pig) Pigments. (*) *p* < 0.05; (**) *p* < 0.01, Wilcoxon signed-rank tests.

The bioassays established that the crude methanol extract of both *Helleborus* spp. leaves stimulated larval feeding activity (*p* < 0.01), thus confirming that we achieved the extraction of the phagostimulant principles from the plant material (Figure 5a).

In the case of *H. foetidus*, these principles were mostly soluble in the *n*-butanol fraction (*p* < 0.01) and to a lesser extend in water (*p* < 0.05) (Figure 5a). The larvae did not show a preference for treatment over control papers when using *n*-hexane or chloroform fractions (*p* > 0.05). Therefore, we selected the *n*-butanol fraction for further subfractionation by column chromatography, as this showed the strongest and most consistent preference with more larvae on treatments in 11 out of 12 dishes.

In the case of *H. viridis* (Figure 5b), all fractions were equally preferred to their controls, with 11 out of 12 replicates showing more larvae feeding on treatments than controls. Due to time limitations, we had to select one fraction to investigate further, so we picked the one where the difference between the mean number of larvae feeding on the treatment and control was greatest, which was the *n*-hexane fraction.

GC-MS analyses (Table 3) showed no differences between the *n*-hexane fractions of *H. viridis* and *H. foetidus* in terms of classes of major identifiable compounds (steroids/triterpenes and pigments), while more clear differences were observed by LC-MS analysis of the *n*-butanol fractions. Among the *n*-butanol fractions of *H. foetidus*, subfraction F was the one and only preferred (Figure 5c); this was then found to contain the saponin compound **1** and sugars. Subfraction D contained compound **1** only, which was also apparently active although short of being significant. Among the *n*-hexane column chromatography subfractions of *H. viridis*, the most preferred was C (Figure 5d); this was then found to contain β-sitosterol (Table 3).

**Figure 6:**
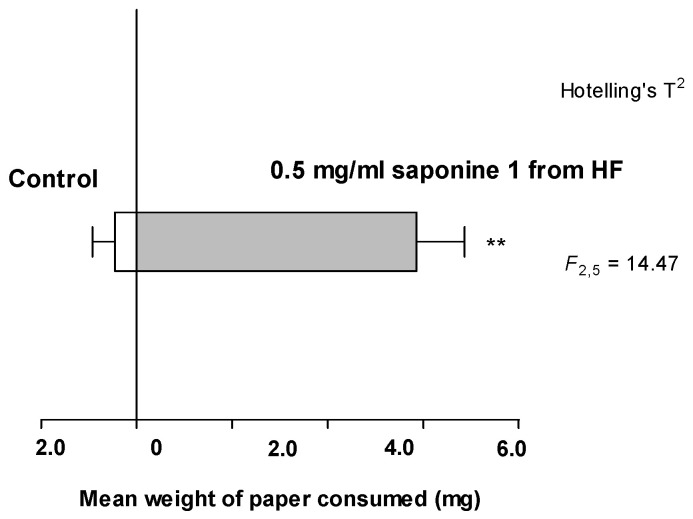
Feeding bioassay of *Monophadnus* species A on paper squares treated with 0.5 mg/mL of compound **1**. (**) *p* < 0.01 and F-distribution were calculated according Hotelling’s T^2^ test. Total weight of paper targets available was on average 80 mg per replicate (40 mg controls, 40 mg treatments), so larvae still had both choices available at the end of the experiment.

Compound **1**—from the active phagostimulant subfraction of the *H. foetidus n*-butanol fraction—was highly active (Figure 6). The saponin is also sequestered and responsible—at least in part—for the deterrence of the larval haemolymph [9]; thus, it is a multifaceted component in the *Monophadnus–Helleborus* system. We did not test any of the nutritional compounds identified in the active subfractions of *Helleborus* spp. (β-sitosterol and sugars), as they are known to act as unspecific phagostimulants for many insects [23,24].

### 2.4. HPLC-UV-ESI/MS-MS of Monophadnus spp. Larval Haemolymph and Helleborus spp. Leaf Extracts

**Figure 7:**
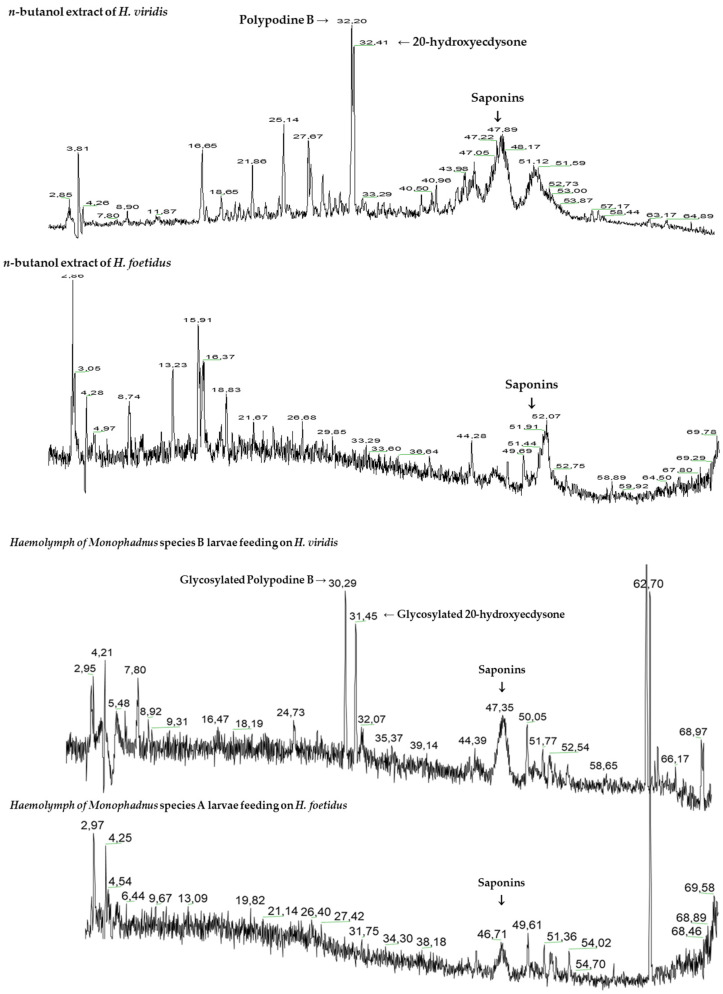
HPLC/MS chromatograms of *Helleborus* spp. *n*-butanol extracts, haemolymph of *Monophadnus* species B larvae feeding on *Helleborus viridis*, and haemolymph of *Monophadnus* species A larvae feeding on *Helleborus foetidus*.

**Figure 8:**
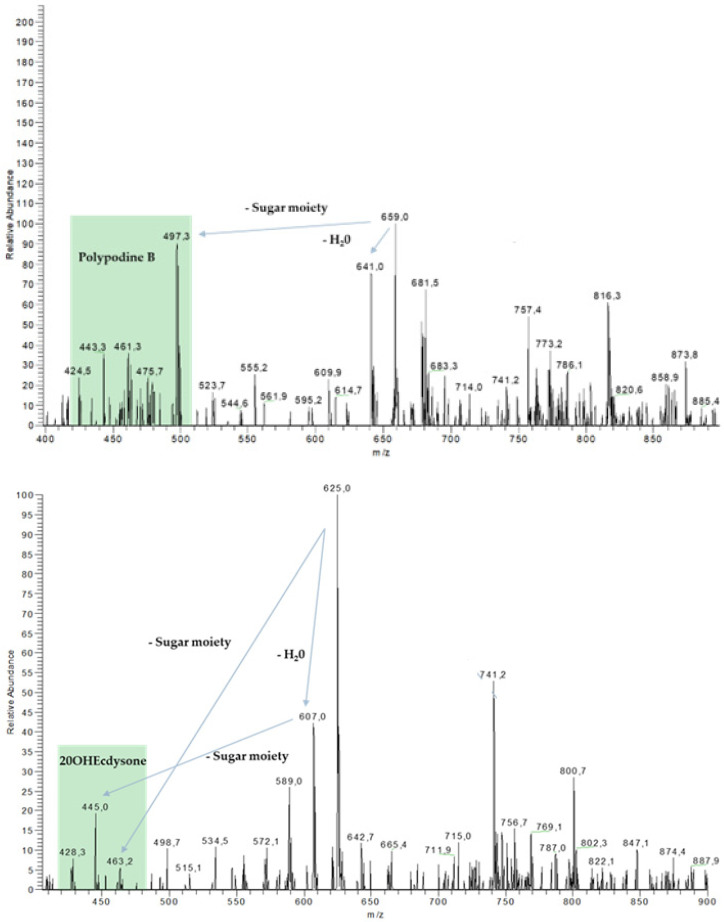
Annotated MS spectra of the ecdysteroids detected in the haemolymph of *Monophadnus* species B feeding on *Helleborus viridis*.

Ecdysteroids were detected and dereplicated with the help of HPLC-UV-ESI/MS-MS (Figure 7 and Figure 8). The methanolic extract from *H. viridis* leaves contained ecdysteroids mainly in form of polypodine B (Rt = 32.20 min) and 20-hydroxyecdysone (32.41 min). These compounds were not detected in the methanolic extract of *H. foetidus* leaves (Figure 7).

Comparative analyses of sawfly haemolymph and plant leaves revealed that more polar derivatives of the plant ecdysteroids (Rt = 30.29 and 31.45 min, respectively) were present in *Monophadnus* species B. The MS spectra of these ecdysteroid derivatives (Figure 8) show at least one loss of a 162 *m*/*z* fragment which may correspond to a hexose moiety, thus accounting for the increase in polarity.

The concentration of ecdysteroid glycosides in *Monophadnus* species B was ca. 10,000-fold higher than their aglycones in the plant in terms of the fresh weight of the samples (Table 4). The amount of ecdysteroid glycosides per mg dry haemolymph resulted to be 0.054 ± 0.030 µmol for polypodine B glycoside and 0.049 ± 0.029 µmol of 20-hydroxyecdysone glycoside (N = 3), achieving 62.70 ± 43.56 µM and 56.75 ± 40.38 µM in the fresh haemolymph, respectively.

### 2.5. Cross-Rearing Experiment

**Figure 9:**
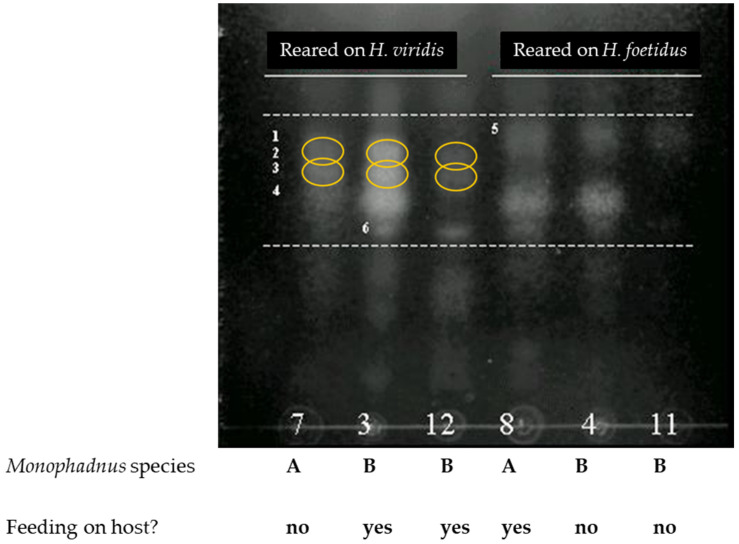
TLC analysis (mobile phase: BAW; reagent: cerium sulphate; picture taken under 254 nm UV light in inversed B/W for visual enhancement) of the haemolymph of *Monophadnus* species A (lanes denoted after sample codes 7 and 8; see Section 4.1) and species B (lanes denoted after sample codes 3, 4, 11, and 12; see Section 4.1) reared either in *Helleborus viridis* or *Helleborus foetidus*. The yellow circles highlight areas where ecdysteroid metabolites can be detected. The small numbers between dashed lines correspond to different metabolites found in that zone.

After the cross-rearing experiment, the glycosylated ecdysteroids could be detected in the haemolymph of *Monophadnus* species A when the larvae were fed on leaves of *H. viridis* only. Conversely, rearing *Monophadnus* species B on its non-native host plant *H. foetidus* resulted in no traces of glycosylated ecdysteroids in their haemolymph (Figure 9). These observations rule out an endogenous synthesis of ecdysteroids from other steroidal precursors found in the plant.

### 2.6. Ecdysteroid Levels in Repeatedly Collected Haemolymph

The results showed that ecdysteroid concentrations were similar on day 1 and day 2 but significatively decreased on day 3 (Table 5). In parallel, the survival of the larvae also dramatically decreased from day 2 to day 3.

### 2.7. Feeding Deterrent Effect on Ants of Haemolymph and Sequestered Plant Metabolites

All samples were endowed with significant deterrent effects at a concentration of 0.8 mg/mL. Moreover, the isolated compound retained its activity even at concentrations ten times lower (Table 6).

## 3. Discussion

### 3.1. Entomological Considerations

Species names such as *M. monticola* (Hartig, 1837), *M. longicornis* (Hartig, 1837), and *M. latus* (Costa, 1894) have been used to describe species feeding on *Helleborus* spp. [25,26,27]. According to the current taxonomy of the genus, these sawflies belong to *M. taegeri* (Lacourt and Noblecourt, 2020) [28,29], but the actual taxonomy of this genus appears to be more complex [28]. The *Monophadnus* larvae we collected from the two different *Helleborus* species showed very clear and consistent morphological (Figure 1) and behavioural differences (species A being nocturnal, B diurnal). We therefore have grounds for considering our two *Monophadnus*, labelled A and B, to be separate species, but the nomenclature remains to be determined by a wider revision of the genus *Monophadnus* which contains ca. 25 species worldwide [30].

### 3.2. Comparative Analysis of All Major Secondary Plant Metabolite Classes in Helleborus and Monophadnus Species

A preliminary comparative chemical screening of insect and plant materials using different instrumental chromatography techniques showed furostanol saponins present in both host-plant species and in the haemolymph of the two sawfly species (Table 1), which agrees with our previous work [9]. Previous works reported on the occurrence of phytoecdysteroids in certain Ranunculaceae species only [14]. Indeed, ecdysteroids were detected in *H. viridis* and the haemolymph of *Monophadnus* larvae reared on its leaves but not in *H. foetidus*. Fatty acids that were present in the plant samples are virtually absent in the haemolymph of the larvae. The presence of other ubiquitous dietary compounds such as sugars, phenolic compounds, and β-sitosterol in haemolymph did not show a consistent pattern.

Therefore, saponins and ecdysteroids were shortlisted as potential feeding stimulants (phagostimulants) for the sawflies and/or as a chemical defence mechanism. A further strong candidate for these activities was ranunculin (compound **2**, Figure 2). This hypothesis is biologically significant as this compound has been reported to occur in all five host-plant genera for *Monophadnus* species—*M. aequalis* (MacGillivray, 1908) feeds on *Anemone* spp., *M. alpicola* (Benson, 1954) on *Pulsatilla* spp., *M.* monticola (Hartig, 1837) on *Helleborus* spp. and *Ranunculus* spp., *M. nigriceps* (F. Smith, 1874) and *M. spinolae* (Klug, 1816) on *Clematis* spp., and *M. pallescens* (Gmelin, 1790) on *Ranunculus* spp. [31,32,33]—plus in three other genera of the Ranunculaceae, whereas this compound is not detected in seventeen other Ranunculaceae genera [34]. Thus, host-plant use by *Monophadnus* sawflies is significantly associated with the occurrence of ranunculin in the plants (*p* = 0.001, Fisher exact probability test; N = 25 Ranunculaceae genera). Unfortunately, the reactivity and instability of this compound’s derivatives precluded feeding assays at room temperature lasting for hours. Our analysis of essential oils extracted by hydrodistillation from the host plants revealed a significant difference in the compound’s concentration between *H. viridis* and *H. foetidus* (trace amounts vs. 0.6%, respectively) (Table 2). Consequently, it is challenging to definitively attribute a chemoecological role of ranunculin for *Monophadnus* larvae.

### 3.3. Phagostimulants and Chemical Defence in the Two Helleborus Species with Monophadnus species B Larvae

Feeding experiments were carried out to find out the plant metabolites responsible for the selective preference of *Monophadnus* species B larvae for *Helleborus foetidus* and *H. viridis* leaves (Figure 5).

When offered the crude extract and different polarity fractions from *H. foetidus*, the feeding of the larvae was significantly stimulated by the *n*-butanol extract and water to a lesser extent. A subsequent assay was conducted with its column chromatography subfractions. Only one showed significant stimulation, which contained compound **1** and sugars. The latter are nutritional compounds known to act as unspecific phagostimulants for many insects [23,24], so only compound **1** was further assayed resulting to be endowed with significant phagostimulant activity (Figure 6). This adds another chemoecological role to this saponin which was already reported as a sequestered deterrent by *Monophadnus* spp. larvae [9].

When offered the crude extract and different polarity fractions from *H. viridis* leaves, the larvae were stimulated by all of them with the same statistical significance. The *n*-hexane fraction was selected for further fractionation, as it was marginally more active. Only one subfraction showed significant stimulation and contained β-sitosterol, a nutritional compound known to act as an unspecific phagostimulant for many insects [23,24]. However, we selected it for further deterrence assays, and it showed an activity at 0.8 mg/mL (Table 6).

Besides compound **1** [9], the characteristic γ-lactones of Ranunculaceae *R*-type species (Figure 3) and ecdysteroids (Figure 4) are potentially also involved in the chemical defence of the larvae due to their blistering and moulting effects, respectively [35,36]. However, the short life and extreme reactivity of the γ-lactones pose technical challenges for their experimental handling. Ecdysteroids are analogues of invertebrate steroid hormones which interfere in the process of insect moulting and are also known to be a deterrent to invertebrate herbivores [37]. Ecdysteroids occur in plants as a complex mixture, but the major compounds are usually ecdysone, its 20-hydroxylated derivative—also known as ecdysterone, polypodine A, or β-ecdysone—(compound **5**; Figure 4) and polypodine B (compound **6**; Figure 4) [38]. Several *Helleborus* species can contain the two latter compounds, but *H. foetidus* belongs to those few evergreen *Helleborus* species not synthesising ecdysteroids in detectable quantities [37].

The results from the cross-rearing experiments (Figure 9) followed by targeted analyses revealed that larvae of both *Monophadnus* species can sequester the ecdysteroids by a glycosylation process if the aglycones are present in the offered *Helleborus* leaves (Figure 7 and Figure 8).

Therefore, the larvae of the two *Monophadnus* species primarily feeding on *H. foetidus*—which does not synthesise ecdysteroids—are tolerant to ecdysteroids when these are experimentally added to the diet, and the larvae are able to both glycosylate and accumulate these compounds in their haemolymph when feeding on *H. viridis* together with compound **1**.

### 3.4. Role of Ecdysteroids in Chemical Defence of Monophadnus spp. Larvae

The HPLC-UV/DAD-MS analyses of both plant extracts and insect haemolymph in specific conditions for the detection of both ecdysteroids and saponins revealed that two aglycones (20-OH-ecydsone and polypodine B) present in the plant extracts are present in the larvae haemolymph in the form of glycosides (Figure 7 and Figure 8). They also showed the presence of compound **1** in all samples. The concentration of ecdysteroid glycosides in *Monophadnus* species B was ca. 10,000-fold higher than their aglycones in the plant (Table 4). Thus, it is likely that this sawfly species glycosylates the ecdysteroids and maintains high levels of these compounds in the haemolymph, which suggests an active sequestration process. These levels were maintained after at least one simulated attack (Table 5). The biological activities of many glycosylated ecdysteroids are significantly lower than those of their corresponding aglycones, likely because the presence of this substituent impairs the interaction with the ecdysteroid receptor(s) [39]. Therefore, glycosylation may help to avoid interference of the ecdysteroids with the insect’s hormone balance, but this would need further experiments to be confirmed. Anyhow, these ecdysteroids seem like a facultative addition to the defence of *Monophadnus* larvae, nevertheless representing a useful contribution to larvae protection which is primarily based on the presence of compound **1**.

In our bioassays, we found a significant deterrent activity to ants using 0.08 mg/mL (166 µM) of 20-hydroxyecdysone and 0.8 mg/mL of the haemolymph (equivalent to ca. 4–10 µM of glycosylated ecdysteroids) both with similar deterrent activity (Table 6). If we suppose that the glycosides are fully hydrolysed within the ant and that polypodine B is as active as 20-hydroxyecdysone, then the haemolymph seems to be 16–41 times more active than what the ecdysteroids account for, which may be due to the contribution of the furostanol saponins acting as a primary defence deterrent achieving 70% of deterrence at 0.08 mg/mL (72 µM) [9]. Whether ecdysteroids and saponins act additively or synergistically requires further experiments.

The concentration of ecdysteroids in fresh haemolymph was maintained after the first simulated attack but decreased more than 60% after the second one during the survival test (Table 5). This could be due to either a decrease in the mass of ecdysteroids in haemolymph or a dilution phenomenon. The concentrations of ecdysteroids decrease both in terms of their μM and μmol/mg haemolymph dry weight, thus suggesting the first phenomenon. The experiment also suggests that the larvae survived the loss of significant amounts of haemolymph if provoked once but not twice on two consecutive days. The reduced fitness of the larvae may then also affect sequestration of plant secondary metabolites.

Predatory insects with biting–chewing mandibles constitute the type of predators to which the easy bleeding of deterrent haemolymph is most effective, but vertebrate predators such as birds also seem to be deterred [40]. In the field, workers of the ant *Lasius niger* L. were observed to retreat immediately after biting into a larva of *Monophadnus* species B, thereby getting into contact with its haemolymph; the ants subsequently cleaned their mandibles for several minutes [9]. Thus, testing the sawfly haemolymph on ants in laboratory bioassays reflects predator–prey interactions that occur under natural conditions.

### 3.5. Future Research and Concluding Remarks

We here demonstrated for first time that glycosylated ecdysteroids play an adjuvant role in the chemical defence of *Monophadnus* spp. larvae and that compound **1** is the main phagostimulant and more potent deterrent compound present in both the larvae’s host plant and their haemolymph. In *Monophadnus* spp. larvae, these ecdysteroids are not stored in specialised glands like in other insects [41] but freely circulate in the larval haemolymph [42]. Therefore, glycosylation seems to protect the insect from their deleterious effects by increasing the water/aqueous medium solubility of the parent compounds, thus facilitating their accumulation in the haemolymph whilst diminishing their ability to cross membranes as well as diminishing their affinity to ecdysteroid receptors [39].

The glycosylation of dietary ecdysteroids by the larvae should rely on the existence of enzymes responsible for the process. Where these are located in *Monophadnus* spp. larvae and what their specificity is are matters that warrant further research. In other insects, “non-targeted” UDP-glucosyltransferases are responsible for the detoxification and elimination of a wide range of endogenous and exogenous compounds including toxins [43]. It is also well known that “ecdysteroid-targeted” enzymes are present in baculoviruses. The baculovirus enzyme ecdysteroid UDP-glucosyltransferase (EGT) disrupts the hormonal balance of the insect host by catalysing the conjugation of ecdysteroids, the moulting hormones, with the sugar moiety from UDP-glucose or UDP-galactose [43]. The natural balance between insects and viruses allows many infected insects to bypass the feeding arrest preceding moulting, leading to larger size before eventual ecdysis [44]. Only future research will unveil if *Monophadnus* species have their own enzyme—targeted or “non-targeted”—or if they have harnessed a symbiosis with EGT^+^ baculoviruses to handle the high concentrations of ecdysteroids present in their diet.

Another point that warrants further scrutiny is the clarification of the insect taxonomy. As discussed in the introduction, we have morphological evidence that suggests that *Monophadnus* species A and B are two separate species. Our work demonstrated that there is a clear difference in their behavioural responses in feeding experiments, with species A being nocturnal and species B being active in their behavioural responses during the day. Both can feed on both *H. viridis* and *H. foetidus* species, able to sequester ecdysteroids wherever they are present in the diet as well as compound **1**. The physiological maintenance of distinct sequestration processes between closely related species is known from sawflies of the genus *Athalia* [42] and, more generally, from other insects such as leaf beetles (Chrysomelidae) [41]. Deterrent assays combining ecdysteroid glycosides and saponins in specific quantities should be carried out to prove if both types of compounds act in synergy towards defending the larvae against predators.

Overall, our results corroborate the chemoecological role of compound **1** in *Monophadnus* species reared on *Helleborus* species as both a feeding stimulant and the main chemical deterrent against predators described in our previous publication [9]. Our present study adds new layers of complexity to the easy-bleeding phenomenon by demonstrating the facultative contribution of dietary plant ecdysteroids to the chemical defence system of *Monophadnus* larvae already based on the occurrence of compound **1**.

## 4. Materials and Methods

### 4.1. Insect and Plant Samples

Larvae of two *Monophadnus* species were collected from several sites in Switzerland on *Helleborus foetidus* and *Helleborus viridis* (Table 7). In accordance with our previous publication [8], we designated *Monophadnus* species A as the one naturally feeding on *H. foetidus* and *Monophadnus* species B as the one naturally feeding on *H. viridis* (Figure 1). Voucher specimens of insect samples used in this study are kept in the collections of the Senckenberg Deutsches Entomologisches Institut (Müncheberg, Germany) and the Royal Belgian Institute of Natural Sciences (Bruxelles, Belgium).

In the laboratory, the larvae were fed fresh leaves of their respective host plants. Haemolymph droplets were collected in glass capillaries after gently piercing the larval integument with forceps. The haemolymph was suspended in ethanol and stored at −20 °C in the dark. Each ethanolic extract was sonicated in an ice bath for 15 min and then vortexed for 1 min. After centrifugation (5 min, 4000 rpm), the supernatant was transferred to a new vial and the precipitate re-extracted twice with methanol under the same conditions. The combined supernatants were dried under N_2_ and subsequently re-dissolved in methanol up to a concentration of 1 to 3 mg/mL.

Full-grown leaves of *H. foetidus* and *H. viridis* were collected in the same areas where *Monophadnus* species A and B larvae were found, respectively, and identified by Alison M. Barker (vouchers deposited at CABI Center, Delémont, Switzerland).

### 4.2. Chemicals

General reagents and solvents of the highest quality were sourced from Sigma-Aldrich (Saint-Louis, MO, USA) unless otherwise stated. The furostanol saponin (25*R*)-26-[(α-L-rhamnopyranosyl)oxy]-22α-methoxyfurost-5-en-3β-yl-*O*-β-D-glucopyranosyl-(1→3)-*O*-[6-acetyl-β-D-glucopyranosyl-(1→3)]-*O*-β-D-glucopyranoside (compound **1;** Figure 2) was obtained as described by Prieto et al. [22].

Protoanemonin (compound **3**; Figure 3)—a product of the hydrolysis of the naturally occurring ranunculin (compound **2**; Figure 3)—was synthesised according to Grundmann and Kober [45], as follows. Angelica lactone (5.7 g) was dissolved in CS_2_ (6.25 mL), and bromine (2 mL) was added dropwise at −20 °C until the solution became colourless. The solution was dried under vacuum, and the residue was dissolved in 40 mL ether. A trace of hydroquinone was added to prevent dimerisation. Then, two equivalents of quinoline (14 g), a tertiary base, were added dropwise at −20 °C, and the reaction mixture was stored overnight at the same temperature. The next day, the excess of quinoline was extracted from the reaction mixture with acidic water; the organic layer filtrate yielded a solid consisting of quinoline hydrobromide. The residue was washed thoroughly with the solvent, which was subsequently eliminated in a rotavapor under low-pressure conditions. The liquid residue was further distilled in a rotary bulb-to-bulb device, and the fraction boiling between 65 °C and 80 °C at 12 torr was collected in a cool trap. Subsequent vacuum distillation yielded the pure compound (bp 68 °C, 8 torr). ^1^H-NMR (CDCl_3_) of the oily, yellow compound corresponded to the data reported in the literature for protoanemonin [35], with traces of anemonin, a product of protoanemonin dimerisation (compound **4**; Figure 3).

The ecdysteroids 20-hydroxyecdysone (compound **5**; Figure 4)—also known as edcysterone, polypodine A, or β-ecdysone—and polypodine B (compound **6**; Figure 4) were sourced from Scitech (Prague, Czech Republic).

### 4.3. Plant Extraction

Freshly collected plant material was chopped and macerated in methanol at room temperature for one week. After concentration under reduced pressure, the crude extract was suspended in methanol 10% *v*/*v* aq. and successively partitioned with *n*-hexane, chloroform, and *n*-butanol to obtain four corresponding fractions. After removing the solvent in a rotavapor, each fraction was freeze-dried to remove any traces of water, the residue was dissolved in methanol (HPLC grade) and centrifuged, and the clear supernatant was used for analysis.

### 4.4. Hydrodistillation and Analysis of Essential Oils from Plant Samples

Fresh leaves (40–60 g) of each *Helleborus* species were collected and subjected to hydrodistillation in a Clevenger apparatus for 2.5 h to obtain the essential oil. The samples were centrifugated (14,000 rpm, Eppendorf centrifuge), and the clear supernatants were stored at −80 °C.

The essential oils were dissolved in hexane and analysed by GC/EIMS with a Varian CP-3800 gas chromatograph equipped with a DB-5 capillary column (30 m × 0.25 mm; coating thickness 0.25 µm) and a Varian Saturn 2000 ion mass detector (Varian, Inc., Walnut Creek, CA, USA). Analytical conditions: injector and transfer line temperatures of 220 °C and 240 °C, respectively; oven temperature programmed from 60 °C to 240 °C at 3 °C/min; carrier gas helium at 1 mL/min; injection of 0.2 μL (10% hexane solution); and split ratio 1:30. The identification of the constituents was based on comparison of the retention times with those of authentic samples as well as on computer matching against NIST98 [46] and Adams [47] libraries’ spectra and a home-made library mass spectra from pure substances.

### 4.5. Comparative Analytical Screening of Plant and Insect Samples

A preliminary screening of the samples of larval haemolymph and crude leaf extracts was performed by thin-layer chromatography using *n*-butanol–acetic acid–water (4:1:5) as the mobile phase and silica gel F_254_ as the stationary phase (Merck, Darmastadt, Germany) and GC-EIMS in a Thermo Quest Trace GC 2000 chromatograph (Thermo Finnigan, San Jose, CA, USA) coupled to a Thermo Finnigan Trace MS mass spectrometer, equipped with a split–splitless injector and a Thermo Finnigan AS 2000 autosampler. A fused silica capillary column (DB-XLB, 15 m × 0.25 mm) was used. Samples were injected (1 µL) in split mode (1:95). The injector was heated to 320 °C, while the transfer line was set at 350 °C and the source temperature at 250 °C. The temperature profile started at 50 °C, followed by a 10 °C/min ramp to 320 °C which was held constant for 15 min, and finally a 10 °C/min ramp up to 360 °C, the thermal maximum of the column. EIMS spectra were acquired in scan mode in a 50–650 *m*/*z* range. The identification of individual constituents was achieved by matching mass spectral data with those held in the NIST98 [46] and a home-made library mass spectra from pure substances.

### 4.6. Targeted Analysis of Ecdysteroids in Plant and Haemolymph Extracts

High-performance liquid chromatographic–photodiode array detection–electrospray ionisation–mass spectrometric (HPLC-UV/PDA-ESI/MS) analyses were performed using a Thermo Finnigan Surveyor LC pump and a Thermo Finnigan Surveyor Autosampler, coupled to a Thermo Finnigan Surveyor PDA detector and a Thermo Finnigan LCQ Advantage ion trap mass spectrometer equipped with Xcalibur 3.1 software (Thermo Finnigan, San Jose, CA, USA). Analyses were performed using a µ-Bondapack C-18 column, 30 cm × 3.9 mm i.d. (Waters Corporation, Milford, MD, USA). We used the step gradient developed by De Combarieu [48] with 0.01% aqueous formic acid (A) and acetonitrile (B) as solvents: 0–20 min, 85–75% A, 15–25% B; 20–40 min, 75–55% A, 25–45% B; 40–55 min, 55–20% A, 45–80% B; 55–65 min, isocratic 20% A, 80% B. The flow rate was 1.0 mL/min. The column was at 23 °C, and the UV channels were set to 246 and 200 nm.

The ESI/MS conditions were positive ion mode, source voltage 4.5 kV, sheath gas (N_2_) at flow rate 60 au, curtain gas (N_2_) at 9.00 au, source current 80 A, capillary voltage 3 V, and capillary temperature 280 °C. Full scan spectra were obtained in the positive ion mode, with a scan time of 1 s from *m*/*z* 250 to 1400. The ion trap was running in automatic gain control with a maximum injection time of 200 ms. For the MS^2^ analyses, the most intense molecular ions were isolated with a width of 1 *m*/*z* unit and fragmented by using an activation amplitude of 25%.

Quantitative analyses were performed using both MS and UV data in parallel. Aliquots of four different concentrations of standards (0.01 to 0.25 mg/mL) were injected into the analytical system, eluted, and monitored with the UV-ESI/MS detectors under the conditions detailed above. Injections of plant extracts and standards were performed in triplicate, on three different days. The response of the ESI/MS detector to different concentrations of ecdysteroids was linear in the range from 0.01 to 0.25 mg/mL (*r* = 0.9998). Haemolymph was injected in duplicate, and three of such analyses were performed on different days. Peak areas were integrated and related to the amount of injected external standard.

### 4.7. Feeding Bioassays

In a “cross-feeding experiment”, larvae of the two *Monophadnus* species were reared on leaves of their non-native host plants as per Table 1: *Monophadnus* species A (sample code 7, Table 7) on *H. viridis* and larvae *Monophadnus* species B (sample codes 4 and 11, Table 1) on *H. foetidus*. This showed that the two sawfly species accepted both plants as feeding hosts with over 70% survival on both. Preliminary experiments to find a methodology for feeding trials discovered that while *Monophadnus* species B was active in the daytime and we were able to follow its behaviour in trials over time, species A was nocturnal, and its lack of activity made it unsuitable for a behavioural trial. As *Monophadnus* species B accepts and grows well on both *Helleborus* species, we chose to use this species for our bioassays.

The phagostimulant activity of plant extracts and isolates in *Monophadnus* species B larvae was investigated in a series of replicated bioassays in which groups of larvae were placed in a dish with a choice of paper squares with a single test solution or a control, and feeding behaviour was monitored. For the initial experiments with *H. viridis* and *H. foetidus* extracts, we tested 12 replicates of the whole extract plus 12 replicates of each fractional extract (made in sequence from non-polar to polar solvents: hexane, chloroform, butanol, water, dried, and redissolved in methanol). We used 10 larvae per dish with the *H. viridis* trials and 9 per dish with *H. foetidus* (fewer were available); each larva was only used once. The larvae were offered a grid of four 1.5 × 1.5 cm squares of filter paper in sealed dishes. All squares were treated with 20 μL of a 1 M sugar solution (without which larvae would not feed on paper); two squares were subsequently treated with 20 μL of extracts or isolates in different concentrations and the other two squares with solvent only (control). A scoping bioassay with different concentrations of whole-leaf extract showed that we obtained a significant feeding response from larvae using 0.25 g fresh weight of leaf material per mL of methanol. We therefore used this as our base concentration, having established a fresh weight-to-dry weight equivalency, and knowing the dry weight proportions of our fractional extracts made up all our test solutions in relation to this.

The number of larvae feeding on control and treatment squares in each dish was visually checked and recorded every 1.5 h for 6 h. Our scoping bioassay had suggested that feeding responses were maximised at 6 h but that this was before the surface of the paper targets had been significantly consumed. Data from the 6 h readings were processed by the Wilcoxon signed-rank test to assess whether feeding activity was significantly higher on extract-treated papers than controls for each treatment at each time—this ranks the difference in numbers between extract and control and tests for a consistent pattern of preference between the two.

Subsequently, we repeated this experimental protocol after the subfractionation of the most active fraction of the *H. viridis* (*n*-hexane) and the *H. foetidus* (*n*-butanol), testing 12 replicates of each subfraction in the same way. We had fewer larvae available for this so were only able to use 6 or 7 larvae in each set of replicates. Again, subfraction concentrations were made by dissolving the dried subfraction in methanol to a concentration equivalent to 0.25 g whole-leaf extract per mL methanol.

To test the isolated saponin (compound **1**, 0.5 mg/mL in methanol), we used 8 larvae per dish but were only able to run 7 replicates. We were able to measure the feeding activity more precisely in this experiment by weighing the dry treated and control filter paper squares before and after the experiment and analysing the weight loss for treatments and controls per dish. As weight is a continuous measure, we analysed the average weight loss using a Hotelling’s T^2^ test.

### 4.8. Larval Survival and Concentration of Ecdysteroids in a Repeatedly Collected Haemolymph Assay

In the laboratory, 18 sham procedure control group larvae and 18 test larvae of *Monophadnus* species B were daily fed fresh leaves of *H. viridis*, weighed, and checked whether they were alive or dead. Haemolymph droplets from larvae of the test group only were collected in glass capillaries after gently piercing the larval integument with forceps once a day over three days. The haemolymph was suspended in ethanol and stored at −20 °C in the dark. Each ethanolic extract was sonicated in an ice water bath for 15 min; the supernatant was vortexed vigorously for 1 min and centrifuged (5 min/4000 rpm). Then, the supernatant was transferred to a new vial and dried under nitrogen steam. The precipitate was extracted twice with methanol under the same conditions. The final residue was re-dissolved with methanol up to a concentration of 1–3 mg/mL.

### 4.9. Deterrent Activity of the Sawfly Haemolymph and Plant Metabolites against Myrmica rubra Workers

The deterrent activity of the sawfly haemolymph and the standard ecdysteroids was assessed by a bioassay slightly adapted from Schaffner et al. [49]. Forty *Myrmica rubra* L. workers were placed in a Petri dish (9.5 cm diameter), in which they were offered a choice between 50 μL of a control solution (sucrose 0.1 M) and 50 μL of a test solution. The test solution was sucrose 0.1 M containing 8 mg dry haemolymph or 0.8 mg purified plant secondary metabolite/mL solution. Ten-fold dilutions were then tested in the same way (0.8, 0.08, and 0.008 mg/mL). The test was replicated twelve times per concentration. The bioactivity was calculated according to the following formula: deterrence rate (%) = (C − T)/(C + T), where C and T are the numbers of ants feeding on the control and test solution, respectively.

Crude haemolymph was extracted in ethanol and filtered on celite powder, the solvent was evaporated under a stream of N_2_, and the dried extract was then re-dissolved in sucrose 0.1 M. Standards of 20-hydroxyecdysone, ecdysone, and β-sitosterol (min. 95%) were dissolved first in ethanol, then in sucrose 0.1 M (in volumes of 5 and 95%, respectively). Preliminary tests comparing sucrose 0.1 M with ethanol in sucrose 0.1 M (1:19) gave no difference in feeding by *M. rubra* workers.

### 4.10. Statistical Analyses

Data were collected and pre-processed in MS Excel v. 21 (Microsoft, Redmon, WA, USA). Linear regressions, interpolations, and Student, ANOVA, and post-ANOVA tests were conducted with the Instat v.3 software (GraphPad, San Diego, CA, USA). Wilcoxon signed-rank tests were calculated using the online Vassarstats tool [50].

## Figures and Tables

**Table 1:** Presence of different classes of phytochemicals in *Monophadnus* spp. haemolymph as compared with their *Helleborus* spp. host-plant species.

Instrumental Technique	Class of Metabolites	*Helleborus foetidus*	*Monophadnus* Species A	*Helleborus* *viridis*	*Monophadnus* Species B
GC-MS	Fatty acids	+++	-	+++	-
	Phenols	-	-	-	++
	Sugars	-	+	-	-
	Sterols	+	+++	+	-
HPLC-UV-MS	Ecdysteroids	-	-	+	+
	Saponins	+	+	+	+

(+, ++, +++) Relative amounts detected; (-) not detected.

**Table 2:** Composition of the essential oil of *Helleborus* spp. leaves analysed by GC/EIMS.

RT ^a^	LRI ^b^	Compound	Percentages ^c^	
			*H. viridis*	*H. foetidus*
3.73	862	(*E*)-2-Hexenal	1.62	0.61
4.29	882	Protoanemonine	traces	0.56
9.03	1041	Benzeneacetaldehyde	traces	0.15
11.34	1104	Nonanal	-	0.48
15.00	1186	3,9-Epoxy-*p*-mentha	-	0.13
15.26	1197	α-Terpineol	-	0.06
16.35	1235	β-Cyclocytral	-	0.08
16.94	1260	*cis*-Carveol	-	0.07
22.29	1373	Naphtalene-1,2-dihydro-1,4,6-trimethyl	-	0.08
27.29	1449	(*Z*)-Ethylcinnamate	-	0.13
27.75	1489	β-Ionone	-	0.10
28.93	1509	β-Bisabolene	-	0.06
44.76	1873	Long-chain acid	-	0.08
47.04	1992	Ethyl palmitate	-	0.97
56.54	2301	Tricosane	-	0.50
		**% Identified**	**1.62**	**5.77**

(^a^) Retention time (RT, in decimal minutes). (^b^) Linear retention indices (DB-5 column). (^c^) Percentages obtained by FID peak-area normalisation. (-) Not detected.

**Table 3:** Major phytochemical classes identified in the fractions and subfractions tested for phagostimulant activity.

Fractions	*Helleborus viridis*	*Helleborus foetidus*
*n*-Hexane	β-sitosterol ^1^ and pigments ^2^.	β-sitosterol ^1^ and pigments ^2^.
*n*-Butanol	Furostanol saponins ^3,4^, ecdysteroids ^3^, phenols ^4^, and anemonin ^1^.	Furostanol saponins ^3,4^, phenols ^4^, and anemonin ^1,4^.
**Subractions**		
C	β-sitosterol ^2^.	
F		Compound **1** ^2,3^ and sugars ^2^.

^1^ GC-MS; ^2^ TLC; ^3^ HPLC-MS; ^4^ isolation [21,22].

**Table 4:** Concentration of ecdysteroids in the methanolic extract of *Helleborus* spp. leaves (N = 5) and in larval haemolymph of *Monophadnus* species B (N = 2) feeding on *Helleborus viridis*. Values (mean ± SD) are expressed in µmol compound/g fresh weight of sample.

	Polypodine B	20-Hydroxyecdysone	Polypodine B Glycoside	20-Hydroxyecdysone Glycoside
*M. species B*	-	-	4.77 ± 2.88	4.36 ± 2.60
*H. viridis*	3.63 × 10^−4^ ± 0.80 × 10^−4^	2.23 × 10^−4^ ± 0.28 × 10^−4^	-	-
*H. foetidus*	-	-	-	-

(-) Not detected.

**Table 5:** Survival of larvae and concentration of ecdysteroids in haemolymph of a sample population (18 larvae on day 1) of *Monophadnus* species B following repeated haemolymph withdrawal.

	Survival in Group		Polypodine B Glycoside		20-Hydroxyecdysone Glycoside	
	Control	Test	mM FW	µmol/mg DW	mM FW	µmol/mg DW
Day 1	100%	100%	93.5	0.087	85.3	0.078
Day 2	100%	89%	92.2	0.084	83.1	0.077
Day 3	94%	23%	38.7	0.028	36.7	0.027

**Table 6:** Feeding deterrent activity of *Monophadnus* spp. larval haemolymph vs. standards against *Myrmica rubra* ant workers.

Test Objects	Concentration (mg/mL Solution)		
	0.8	0.08	0.008
*Monophadnus* species A haemolymph	42% * (79) ^a^	21% ^n.s.^ (109) ^a^	-
*Monophadnus* species B haemolymph	62% * (100) ^a^	−2% ^n.s.^ (127) ^a^	-
20-Hydroxyecdysone	79% * (77)	46% * (71)	14% ^n.s.^ (58)
Ecdysone	41% * (109)	−4% ^n.s.^ (77)	-
β-sitosterol	67% * (66)	6% ^n.s.^ (83)	-

(*) *p* < 0.05, (^n.s.^) not significant, Wilcoxon matched-pairs signed-ranks test. (-) Not tested. (^a^) Data from our previous work [9]. Total numbers of ants feeding on test and control droplets are given between parentheses.

**Table 7:** Larvae of *Monophadnus* spp. collected in Switzerland on *Helleborus* spp.

Monophadnus Species	Sample Code	Larvae (N)	Location	Host Plant	Reared on	Haemolymph (μL)
A	8	5	D	*H. foetidus*	*H. foetidus*	39
A	7	5	D	*H. foetidus*	*H. viridis*	33
B	3	23	C	*H. viridis*	*H. viridis*	203
B	4	9	C	*H. viridis*	*H. foetidus*	63
B	12	6	M	*H. viridis*	*H. viridis*	33
B	11	11	M	*H. viridis*	*H. foetidus*	48

(C) Crocifisso, canton Ticino; (M) Muggio, canton Ticino; (D) Delémont, canton Jura.

## Data Availability

Data are contained within the article.
